# Forage quality and physiological performance of mowed alfalfa (*Medicago sativa* L.) subjected to combined light quality and drought

**DOI:** 10.3389/fpls.2022.1047294

**Published:** 2022-11-22

**Authors:** Chunxia He, Yan Zhao, Yao Wang, Jinfeng Cai, Jun Gao, Jinsong Zhang

**Affiliations:** ^1^ Key Laboratory of Tree Breeding and Cultivation of National Forestry and Grassland Administration, Research Institute of Forestry, Chinese Academy of Forestry, Beijing, China; ^2^ Co-Innovation Center for Sustainable Forestry in Southern China, Nanjing Forestry University, Nanjing, China; ^3^ Henan Xiaolangdi Earth Critical Zone National Research Station on the Middle Yellow River, Jiyuan, China; ^4^ College of Horticulture and Plant Protection, Henan University of Science and Technology, Luoyang, China

**Keywords:** alfalfa, climate change, water deficit, understory sunlight, forage quality, mowing

## Abstract

Alfalfa (*Medicago sativa*) can dwell in water-deficient habitats, where it is difficult to predict dry mass (DM) production and forage quality due to understory transmittance. Mowing is a recommended practice for alfalfa populations under drought, but its effect on forested land receives less attention. In a controlled indoor experiment, we found that drought better reduces shoot DM weight and crude fiber content (CFi) in blue light (33.7% red, 48.5% green, and 17.8% blue lights) than red light (71.7% red, 13.7% green, and 14.6% blue lights). Mowing decreases carbon (C) isotope signature (δ^13^C), CFi, and total C content in shoots but increases their accumulations in DM, nonstructural carbohydrates, and crude fat content (CFa). The results also demonstrated that mown alfalfa has higher starch content when exposed to green light (26.2% red, 56.4% green, and 17.4% blue lights) compared to the other two spectra. Multiple factorial regression indicated that higher soluble sugar content accounted for the increase of CFa and DM weight for CFi. Overall, mowing in blue-light–enriched understory stands is recommended and produces high-forage–quality alfalfa, which can be used as a lowered crude fiber component.

## Introduction

Due to global climate change, warmer temperatures and fluctuant rainfall patterns may have resulted in a decline in meadow productivity (Meng et al., 2019; Xu et al., 2021). Drought is a climate event that can cause extreme interruption for meadows in a wide range of territorial ecosystems on alpine (Xu et al., 2021), montane (Debinski et al., 2010), and bay landforms (Mulhouse et al., 2005). High-quality forage is a fleeting resource, and drought further reduces their chances by interfering with the phenological stage of plant growth (Aikens et al., 2020). Alfalfa (*Medicago sativa*) is a reliable species of legume that provides an abundance of forage for feeding ruminants (Hanly et al., 2020). Natural alfalfa populations are distributed in arid and semi-arid regions (Yari et al., 2014), where droughts generate a limit for the primary production of local plants (Guo et al., 2016b). Alfalfa is frequently subjected to drought threats. However, some genotypes are substantially tolerant to water deficiency (Guo et al., 2016b; Ma et al., 2021). Alfalfa is widely used as a model forage plant to detect genetic mechanisms of drought tolerance (Zheng et al., 2017; Hanly et al., 2020). For practical meaning, the experimental improvement of the forage quality of alfalfa is of high importance. Genetic parameters also confirm the response of forage quality in alfalfa to drought (Lin et al., 2020; Mustafa et al., 2022). However, the ecophysiological response has not received adequate attention.

Mowing is a commonly used practice to restore perennial plant communities under global warming (Meisser et al., 2013; Deleglise et al., 2015). For alfalfa, mowing can stimulate the regrowth of aerial organs by removing grown shoot biomass (Giese et al., 2013; Han et al., 2014). Practical management of mowing and phosphorus addition was shown to further promote the regrowth of aerial organs and restore degraded alfalfa grasslands (Zhou et al., 2019). Mowing can also reduce competition from seasonal weeds in alfalfa communities (Anderson, 2016). Among general agronomic management practices, mowing can reduce dry mass loss in alfalfa hay caused by raking and baling operations (Al-Gaadi, 2018). Additionally, mowing is much more effective in controlling alfalfa aphids than insecticides (Qiaoyan et al., 2015). However, the operational benefits of mowing were mostly reported in trials aiming to improve net primary productivity from alfalfa hays. The current evidence is still not clear regarding the mowing effect on the forage quality of alfalfa. As an operational practice, mowing can be used as a nature-based solution to cope with issues that arise alongside the natural limitations of drought. To our knowledge, it is still unclear whether mowing has a consistent effect on the regrowth of alfalfa under water-deficient conditions.

Studies on alfalfa quality report on the detection of phytochemical parameters under laboratory conditions. However, the imposed effects of drought on alfalfa involve multiple environmental factors and are more complex than synthesized conditions. As a perennial forage, alfalfa dwells in meadowlands (Wang et al., 2009; Guo et al., 2016a), where populations grow in full sunlight. However, alfalfa can also be distributed under canopies of forest trees (Qin et al., 2022). Light is a fatal factor that may limit the growth and development of undergrowth (Zhao et al., 2020; Chu et al., 2021; Song et al., 2022). Shade can systematically reduce biomass production in alfalfa, but the light deficit would not threaten survival in shading up to 50% (Lin et al., 1998; Varella et al., 2001). It has also been found that shade limits the reproduction of alfalfa through the delay of flowering (Lorenzo et al., 2019; Qin et al., 2022). Given that populations at the understory layer are subjected to the sunlight spectrum of transmittance (Wei et al., 2019; Wei et al., 2020), the light spectrum is a determinative factor of illumination condition for plant growth and development (Chu et al., 2021; Zhou et al., 2021a). The spectrum from a light-emitting diode (LED) was identified to promote the production of total phenolic compounds in alfalfa cotyledons compared to the spectra from other types of illuminations (Fiutak et al., 2019). The red LED spectrum was found to reduce total phenolic content in alfalfa sprouts (Kwack et al., 2015). Information is still insufficient to reveal the comprehensive effects of spectrum on forage quality in alfalfa. Current findings were derived from studies that lacked the imposed stress of water deficit.

Crude fiber is a key component in evaluating alfalfa forage quality. Available fiber components include acid detergent fiber (ADF) and nutrient detergent fiber (NDF); both reduce the forage quality of alfalfa hay (Adjesiwor et al., 2017; Mao et al., 2018). The alfalfa stem is the organ that accomplishes more regrowth when mowed or rewatered (Staniak and Harasim, 2018; Kamran et al., 2022). Stems also have higher ADF and NDF concentrations than other organs (Mao et al., 2018; Lemaire and Belanger, 2020). Therefore, the increment of dry mass accumulation in the stems of alfalfa may alternatively be accompanied by the decline of forage quality due to promoted crude fiber production. Crude fat is another key parameter that determines the quality of alfalfa forage. Fat is a concentrated source of energy, and the fattening of animals requires a diet that is dense in digestible energy (high fat and low fiber) (Ball et al., 2001). The increase in crude fat is a good way to evaluate the quality of alfalfa (Hu et al., 2010; Ullah et al., 2016) because drought stress can induce an increase in crude fat (Staniak and Harasim, 2018). It is difficult to predict the effects mowing will have on the crude fiber and fat ratio in alfalfa. Little is known about the involvement of the understory light spectrum on the response of forage quality in alfalfa.

When a C_3_ plant perceives the stress of drought, it will mostly downregulate the discrimination of the heavier carbon (C) isotope (^13^C) against the lighter one (^12^C) (i.e., δ^13^C). The less a plant discriminates between the two isotopes (δ^13^C more positive), the more stomatal conductance is controlled to limit gas exchange, and the greater water-use efficiency (WUE) is increased (Erice et al., 2011; He et al., 2020). The C isotope signature (δ^13^C) was proven to be a sensitive parameter to indicate WUE in alfalfa subjected to drought (Erice et al., 2011). In this study, alfalfa seedlings were raised in a controlled environment where light conditions mimicked the natural condition and drought was imposed by the withdrawal of water. Seedlings were sampled twice, before and after mowing, and sent to the laboratory for determining forage quality and WUE. The goal was to reveal alfalfa’s response to triple treatments of mowing in different light spectra under drought stress. We also aimed to detect the factors that had conjoined contributions to shaping the forage quality of alfalfa. We hypothesized that either mowing or well-watered treatments could promote shoot growth but decrease crude fiber and fat ratios.

## Materials and methods

### Experimental condition and materials

Alfalfa seeds were collected from Nanshan Forest Farm, Jiyuan, Henan, China. Planted alfalfa populations were used to collect seeds from the understory layer of a walnut (*Juglans regia* L.)-alfalfa agroforestry system (34° 56′−35° 04′ N, 112° 22′−112° 32′ E) in southern Taihang Mountain. Seeds were evenly distributed to planting cavities (212 ml; 7 cm × 4 cm × 13 cm, top Ø × bottom Ø × height) in trays filled with growing substrates (peat and perlite, 3:1, v/v). The tray surface was covered by a moist towel, and the moisture (>95%) was maintained every day by spraying with distilled water. When plantlets were germinated, the number of specimens was thinned to leave about 8–10 individuals per cavity to eliminate unnecessary competition. Thinned seedlings were cultured with a nutritional solution adapted from Kim et al. (1991). Solutions contained 1 mM potassium nitrate (KNO_3_), 0.4 mM monopotassium phosphate (KH_2_PO_4_), 1 mM potassium sulfate (K_2_SO_4_), 3 mM calcium chloride (CaCl_2_), 0.5 mM magnesium sulfate (MgSO_4_), 0.15 mM dipotassium phosphate (K_2_HPO_4_), 0.2 mM iron–sodium ethylene diamine tetraacetic acid (Fe–Na EDTA), 14 μM boric acid (H_3_BO_3_), 5 μM manganese sulfate (MnSO_4_), 3 μM zinc sulfate (ZnSO_4_), 0.7 mM ammonium molybdate ((NH_4_)_6_Mo_7_O_2_), and 0.1 Mm cobalt chloride (CoCl_2_).

### Drought treatment

Half of the alfalfa seedlings were cultured with water withdrawal (drought), and the other half were well-watered (control). The water deficit was induced by a 7-day withdrawal of water input. That is, drought-treated seedlings were watered every 14 days, while the controlled seedlings were watered every day. Thus, for alfalfa, drought stress can be induced after 7, 14, or even 21 days of water deficit, but only the 14-day period of drought-induced mostly frequent negative responses (Erice et al., 2011). Because the irrigation seedlings were watered to the pot capacity, the drought treatment and well-watered control resulted in different total volumes of water input. This accords with the total quantity of water used in the water deficit treatments of Erice et al. (2011). The seedlings that received contrasting rates of water were fed with the same dose of total nutrient input. Drought-treated seedlings were fed with nutrients on the same day water was supplied; the dose equaled that which the controlled seedlings received in a week’s time. During the experiment, the temperature was maintained in a range of 17°C and 34°C (night/day), and the relative humidity was maintained at 52%.

### Light spectra exposure

Alfalfa seedlings were raised under artificial LED lighting. Throughout the experiment, seedlings were exposed to a 12-h photoperiod from 08:00 am to 20:00 pm. This amount of time was shorter than that (~18 h/day) used for woody plants (Wei et al., 2020; Gao et al., 2021) because alfalfa has a faster-growing speed and does not need longer photoperiod exposure to promote growth. As alfalfa seeds were collected from an understory population beneath the canopy of a walnut agroforestry system, their lighting environment was simulated from a wide range of spectra that were tested for generated saplings of walnut (Gao et al., 2021). Three types of spectra were tested. The blue-light spectrum contained proportions of photosynthetic photon flux density (PPFD) of 33.7% red (600–700 nm), 48.5% green (500–600 nm), and 17.8% blue lights (400–500 nm). The red-light spectrum contained PPFD proportions of 71.7% red, 13.7% green, and 14.6% blue lights. The green-light spectrum contained PPFD proportions of 26.2% red, 56.4% green, and 17.4% blue lights. The test of ranged spectra was also employed on understory medicinal herbs (Guo et al., 2020; Zhou et al., 2021a; Zhou et al., 2021b).

Spectra were emitted by illuminations from LED panels (0.5 m × 1.2 m, width × length). It was determined that daytime PPFD under forest canopy ranged from 3.60 to 175.67 μmol m^−2^ s^−1^ (Wei et al., 2020). For each generation of walnut saplings, PPFD in touchable space was around 96 μmol m^−2^ s^−1^ (Gao et al., 2021), which fell in the range of PPFD in transmittance of sunlight. Therefore, PPFD was adjusted to be 97.88 μmol m^−2^ s^−1^ 10 cm beneath the LED panel. The LED panels were hung 50 cm over the tray. Three transformers were responsible for controlling the electrical currents of panel diodes. The 200-W transformer accounted for the electrical current adjustment of red-light diodes, and the 135-W transformer controlled that of green- and blue-light diodes.

### Mowing and sampling

The combined treatments of light spectra (*df* = 2) and water deficit (*df* = 1) were replicated three times, each of which was assigned as a tray of alfalfa seedlings. When the maximum height of most alfalfa per tray nearly reached ~50 cm (tips touched the panel), seedlings were mowed to remove all above-ground parts. Seedlings were clipped to mimic mowing about 50 days after sowing, and 58 days later the seedlings were clipped again to harvest for sampling. The mowing treatment was incorporated into the experimental arrangement as a repeated manipulation and did not increase the number of fixed-factor replicates. Most aerial organs were mowed, leaving shoots at a height of about 5 cm, as suggested by Shen et al. (2013). Mowed samples were divided into two halves. One-half of the samples were measured for height and then dried in an oven (70°C) for 72 h. Their dry mass (DM) was measured, and chemical analyses followed. The other half was freeze-dried and used for measuring physiological parameters.

### Parameter determination

Oven-dried samples were ground to pass a 1.0-mm screen. Soluble sugar and starch contents were determined by a colorimetric method (DuBois et al., 1956). A 0.5-g sample was used to calculate colorimetric measurement at 490 nm using an UV-Visible 8453 analyzer (Agilent Inc., San Francisco, CA, USA). Crude fiber and fat contents were determined using the standard methods endorsed by relevant national standards. Crude fiber determination was adapted from the method of SN/T 0800.8-1999 (2000) and crude fat from GB/T 6433-2006/ISO 6492: 1999 (2006). C-isotope discrimination was determined using freeze-dried samples that passed a 1-mm sieve. δ^13^C was determined using a mass spectrometer (Thermo Finnigan, CA, USA) following the equation:


(1) $$\delta^{13}C\left(‰\right)=\left(\frac{R_{Sample}}{R_{Standard}}-1\right)\times 1,000$$

where *R*_Sample_ and *R*_Standard_ are the ratios of ^13^C/^12^C in plant samples and the standard (Pee Dee Belemnite). Total C content was determined by an element analyzer (EA-3000, Boaying Tech., Shanghai, China).

### Statistical analysis

Results were analyzed in a mixed-model analysis of variance (ANOVA), where light spectra and drought treatment were two fixed factors that were replicated three times, and seedlings were sampled twice pre/postmowing. The random placement of trays was designated as a random factor. SAS software (SAS Inc., Charlotte, NC, USA) was used to analyze the data. Factors of water deficit, mowing treatment, and light spectra were combined as a multiple-factorial interaction design. When significant effects were indicated, results were compared across treatments following the Tukey test (*α* = 0.05). To reveal the joint driving forces of ecophysiological parameters, multivariate linear regression was used to regress the contributions of crude fat and fiber contents. Pearson correlation was used to detect relationships between pairs of ecophysiological parameters.

## Results

### Growth and DM accumulation

**Figure 1:**
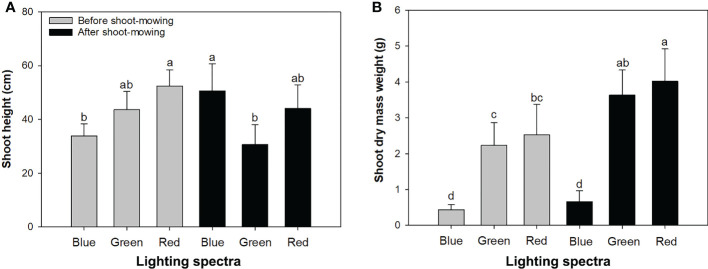
Height **(A)** and dry mass weight **(B)** in mowed alfalfa shoots exposed to varied spectra of blue, green, and red lights. Different lowercase letters indicate significant differences at 0.05 level.

Light spectra had an interactive effect with mowing on height and DM (**Table 1**). Before shoot-mowing, the red-light spectrum induced greater shoot height compared to the blue-light spectrum (**Figure 1A**). Regarding the blue-light spectrum, postmowing seedlings had greater shoot height compared to those which had not yet been mowed (**Figure 1A**). Blue light also induced greater shoot height in the postmowing seedlings compared to green light. Water also had an effect on height and DM. Both height and DM increased in the well-watered plants compared to those under drought conditions (**Table 2**).

**Table 1:** *F*-values from analysis of variance (ANOVA) of shoot-mowing (Mow), light-emitting diode (LED) spectra (Light), moist condition (Water), and their inter- and multicombinations on growth, biomass, and carbon (C) metabolism in alfalfa (*Medicago sativa* L.) seedlings.

Source of variance	*df*	Height (cm)	DMW (g)	Sugar (mg g^−1^ DW)	Starch (mg g^−1^ DW)	Fat^d^ (%)	CFiber (%)	δ^13^C (‰)	Total C (%)
**Mow**	**1**	**0.53**	41.05^***^	12.13^**^	13.87^**^	7.54^*^	9.36^**^	6.26^*^	**3.41**
**Light**	**2**	9.82^***,a^	110.10^***^	8.69^**^	4.10^*^	**1.92**	21.58^***^	9.82^**^	11.59^***^
**Water**	**1**	28.74^***^	32.17^***^	**2.28**	18.63^***^	5.33^*^	9.60^**^	48.18^***^	90.90^***^
**Mow × Light**	**2**	20.42^***^	6.29^**^	**0.26**	8.72^**^	**0.22**	**0.08**	9.08^**^	17.30^***^
**Mow × Water**	**1**	**0.43**	**0.03**	**0.32**	**0.27**	**1.15**	**0.49**	**3.07**	9.85^**^
**Light × Water**	**2**	**0.04**	3.58^*^	**0.96**	**1.12**	**1.54**	**0.58**	**0.31**	**0.14**
**Mow × Light × Water**	**2**	**0.03**	**0.19**	**0.36**	**3.11**	**1.80**	**0.22**	**0.04**	**0.12**

df, degree of freedom; DMW, DM weight; DM, dry mass; Fat, fat content; CFiber, coarse fiber content.

a Significance categories of p-values: ^*^p< 0.05; ^**^p< 0.01; ^***^p< 0.001.

**Table 2:** Drought effect on growth, DM accumulation, carbohydrate metabolism, quality establishment, and C in alfalfa (*Medicago sativa* L.) seedlings.

Seedling parameter	Well-watered	Drought
**Height (cm)**	**48.01 ± 9.09 a**	**37.01 ± 7.76 b**
**DMW (g)**	**2.71 ± 1.43 a**	**1.79 ± 1.08 b**
**Sugar (mg g^−1^ DW)**	**5.93 ± 2.09 a**	**4.30 ± 1.86 a**
**Starch (mg g^−1^ DW)**	**15.00 ± 6.71 a**	**8.94 ± 3.39 b**
**Fat (%)**	**2.76 ± 1.66 a**	**1.76 ± 0.73 b**
**Fiber (%)**	**25.86 ± 4.26 b**	**28.99 ± 3.69 a**
**δ^13^C (‰)**	**−34.99 ± 0.57 b**	**−33.82 ± 0.69 a**
**Total C (%)**	**40.90 ± 1.74 b**	**44.83 ± 1.85 a**

DMW, DM weight.

In the seedlings that were not mowed, exposure to the red-light spectrum resulted in greater shoot DM than in those under the blue-light spectrum, as well as in the mowed seedlings (**Figure 1B**). Mowing increased shoot DM weight in the seedlings exposed to green- and red-light spectra.

**Figure 2:**
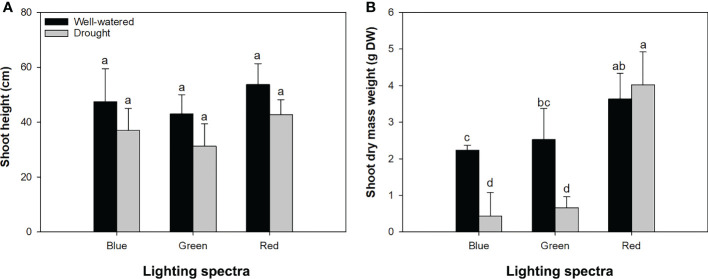
Height **(A)** and dry mass weight **(B)** in alfalfa shoots exposed to contrasting water conditions and varied spectra of blue, green, and red lights. Different lowercase letters indicate significant differences at 0.05 level.

Although watering conditions and light spectra had no interactive effects on shoot height (**Figure 2A**), their interactions had a significant impact on DM weight (**Figure 2B**). Drought-exposed seedlings were depressed to accumulate shoot DM in the blue- and green-light spectra, but water conditions did not change the shoot DM weight in the red-light spectrum (**Figure 2B**).

### Nonstructural carbohydrate accumulation

**Figure 3:**
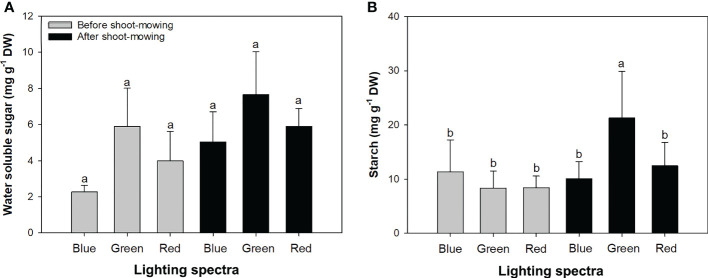
Contents of water-soluble sugar **(A)** and starch **(B)** in mowed alfalfa shoots exposed to varied spectra of blue, green, and red lights. Different lowercase letters indicate significant differences at 0.05 level.

Mowing and light spectra had an interactive effect on soluble sugar and starch concentrations (**Table 1**). Soluble sugar content was increased by 53% after mowing (before mowing, 4.05 ± 1.91 mg g^−1^ DW; after mowing, 6.19 ± 1.85 mg g^−1^ DW). The green-light spectrum resulted in higher soluble sugar content (6.77 ± 2.22 mg g^−1^ DW) compared to the blue- (3.64 ± 1.70 mg g^−1^ DW) and red-light (4.94 ± 1.52 mg g^−1^ DW) spectrums. Starch content was the highest in mowed seedlings exposed to green light (**Figure 3B**). Starch content decreased during the drought treatment, while the change of soluble sugars was not significant (**Table 2**).

### Crude fat and fiber

**Figure 4:**
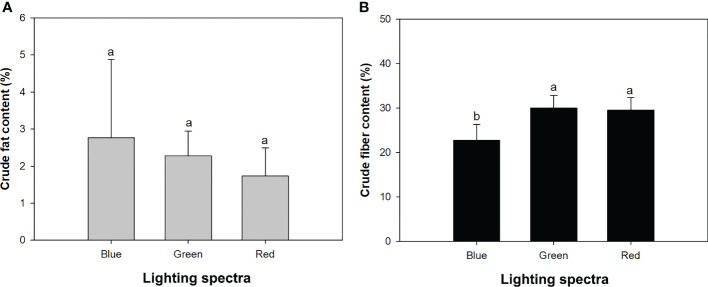
Contents of crude fat **(A)** and fiber **(B)** in alfalfa shoots exposed to varied spectra of blue, green, and red lights. Different lowercase letters indicate significant differences at 0.05 level.

Mowing increased crude fat content from 1.66% ± 0.77% to 2.86% ± 1.59% but decreased crude fiber content from 28.97% ± 3.86% to 25.88% ± 4.11%. The varying light spectra did not significantly affect crude fat content (**Table 1**), which ranged between 1.7% and 2.8% among the three types of spectra (**Figure 4A**). Crude fiber content under the blue-light spectrum decreased by 32% and 30%, compared to that in the green- and red-light spectra, respectively (**Figure 4B**). The drought treatment induced a decrease in crude fat content but an increase in crude fiber content (**Table 2**).

### δ^13^C and total carbon

**Figure 5:**
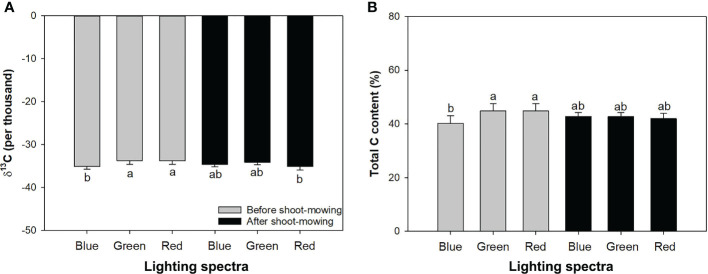
Delta isotope-13 carbon (C) **(A)** and total C content **(B)** in mowed alfalfa shoots exposed to varied spectra of blue, green, and red lights. Different lowercase letters indicate significant differences at 0.05 level.

Mowing and light spectra had an interactive effect on δ^13^C and total carbon content (**Table 1**). Mowing decreased δ^13^C for seedlings exposed to the red-light spectrum, but no variation of δ^13^C was induced by mowing in the blue- and green-light spectra. Before mowing, the blue-light spectrum induced lower δ^13^C compared to the green- and red-light spectra. However, after mowing, the variation of δ^13^C disappeared among spectra (**Figure 5A**). Before mowing, total C content was higher in seedlings subjected to the green- and red-light spectra, but a postmowing difference in total C content disappeared again (**Figure 5B**). The drought resulted in higher δ^13^C (**Table 2**).

**Figure 6:**
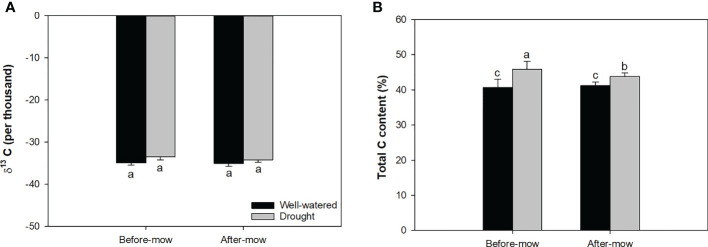
Delta isotope-13 carbon (C) **(A)** and total C content **(B)** in mowed alfalfa shoots exposed to contrasting water conditions. Different lowercase letters indicate significant differences at 0.05 level.

Mowing and water conditions did not result in a significant difference in δ^13^C (**Table 1**; **Figure 6A**). However, the drought treatment increased total C content regardless of whether the shoots had been mowed (**Figure 6B**). Mowing decreased total C content in drought-treated seedlings. Drought increased δ^13^C, fiber content, and total C content but decreased DM and fat content (**Table 2**).

### Driving forces of physiological parameters for forage quality

**Figure 7:**
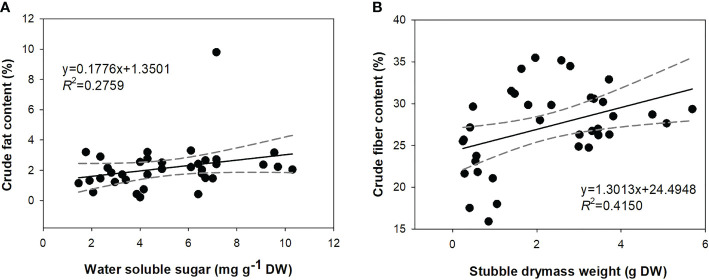
Correlations between water-soluble sugar content and crude fat content **(A)** and shoot dry mass weight and crude fiber content **(B)**. Full lines are fit curves; black color dots are observation values; dashed lines range from 95% confidence bands.

The linear regression model indicated three physiological parameters (shoot DM weight, soluble sugar content, and total C content) that may contribute to the accumulation of crude fat (**Table 3**). However, the only sugar content was estimated to be a driving force for crude fat. DM weight and total C content were screened, and DM was further estimated as a significant parameter. Water-soluble sugar contributed to a positive correlation with crude fat content, which can be shown by a curve with a low slope (~0.18) with the narrowest 95% confidence falling in a range of 4.3−6.1 mg g^−1^ DW. Shoot DM weight positively contributed to crude fiber content, whose 95% confidence was narrowest in a range of 2.1−3.3 g DW (**Figure 7B**).

**Table 3:** Multivariate linear regression of crude fat and crude fiber contents against growth and physiological parameters in alfalfa (*Medicago sativa* L.) seedlings.

Dependent variables	Variables	Parameter estimate	SE	*F*-value	*p*-value
**Crude fat (%)**	**Intercept**	**7.82**	**3.61**	**4.69**	**0.038**
	**DMW (g)**	**−0.31**	**0.18**	**2.82**	**0.1027**
	**Sugar (mg g^−1^ DW)**	**0.30**	**0.12**	**6.62**	**0.015**
	**C (%)**	**−0.15**	**0.08**	**3.07**	**0.0892**
**Crude fiber (%)**	**Intercept**	**3.90**	**10.91**	**0.13**	**0.7229**
	**DMW (g)**	**1.16**	**0.48**	**5.83**	**0.0215**
	**C (%)**	**0.49**	**0.26**	**3.61**	**0.0661**

SE, standard error; DMW, DM weight; Sugar, water-soluble sugar content; C, total C content.

Apart from forage-quality variables, physiological parameters had close relationships (**Table 4**). Shoot DM weight had a positive correlation with soluble sugar content, which further had a positive correlation with starch content. Total C content had a positive correlation with δ^13^C.

**Table 4:** Pearson analysis of the correlation between paired parameters concerning growth, DM accumulation, and physiology in alfalfa (*Medicago sativa* L.) seedlings.

	Height	DMW	Sugar	Starch	δ^13^C	Total C
**Height**	1	0.05126	0.05976	−0.1716	0.26487	0.15239
		0.7666	0.7292	0.317	0.1185	0.3749
**DMW**		1	**0.49806**	0.29455	0.27188	0.15064
			**0.002**	0.0812	0.1087	0.3805
**Sugar**			1	**0.44567**	0.22223	0.12239
				**0.0064**	0.1927	0.477
**Starch**				1	0.14586	−0.06086
					0.396	0.7244
**δ^13^C**					1	**0.3895**
						**0.0189**
**Total C**						1
					

DMW, DM weight; Total C, total C content.

Values in bold font indicate significant correlations in the confidence level of p< 0.05.

## Discussion

### Growth and DM in alfalfa exposed to mowing, drought, and light spectra

We found that mowing can increase shoot DM weight in alfalfa when exposed to different light spectra. Moreover, shoot DM can be promoted by mowing in red- and green-light spectra, but no response was found in the blue light. A field trial also reported an increased DM production in alfalfa following mowing (Al-Gaadi, 2018). The increase in DM production in alfalfa populations resulted from the promotion of the regrowth of shoot parts under field conditions following mowing (Giese et al., 2013; Han et al., 2014). However, under blue light, mowing did not cause any changes in DM but did increase shoot height. DM increment in mowed alfalfa may be due to joint increments in plant organs such as flowers, buds, nodules, and initial shoots (Anower et al., 2017; Fiutak et al., 2019). This can account for the irrelevance of height growth with a null response of DM. Mowing was also found to increase the stem length of *Aralia elata*, a woody species, under plant factory conditions (Wei et al., 2020). Therefore, our first hypothesis can be accepted under the condition of blue-light radiation.

Our drought treatment limited shoot height across all light spectra, but water conditions did not affect DM weight in any of the light spectra. Aranjuelo et al. (2007) also reported that drought depressed growth and DM accumulation in nodules of alfalfa. Some accessions of alfalfa are extremely drought tolerant and showed more shoot biomass when subjected to drought (Anower et al., 2017). The cultivar, however, was not as tolerant. The results endorse parts of our results that the well-watered condition can promote the shoot growth of alfalfa.

It was proven that DM production in alfalfa can be easily modified by changing lighting spectra (Fiutak et al., 2019). Compared to the green-light spectrum, a blue-light–enriched spectrum more efficiently promotes dry matter accumulation in alfalfa’s aerial organs (Fiutak et al., 2019). Regenerated oak saplings also showed greater shoot biomass under blue light compared to their performance under green light (Gao et al., 2021). Our results did not follow the trend of these findings. The blue light resulted in lower shoot DM accumulation compared to both the green- and red-light spectra. This result was not influenced by mowing. In the interaction with water conditions, the red light only increased shoot DM accumulation. In dill (*Anethum graveolens* L.) and lettuce cultivars, red light caused greater DM production than blue light (Fraszczak, 2013; Clavijo-Herrera et al., 2018). The red-light spectrum resulted in greater shoot DM under drought conditions. Therefore, the red-light spectrum can be identified to benefit DM production in shoots.

### Metabolism of nonstructural carbohydrates in alfalfa exposed to mowing and light spectra

Mowing increased starch content in alfalfa as a general effect, especially for those plants under green light. Annicchiarico et al. (2013) reported positive responses of increased starch accumulation in five alfalfa cultivars to a mown environment. They concluded this controls starch degradation. The accumulation of nonstructural carbohydrates is the main support in the regrowth of perennial root shoots (Annicchiarico et al., 2013). Both mowing and the green-light spectrum promoted shoot starch content in *A. elata* (Wei et al., 2020), which depended on its sprouts to regrow its shoots. In annual grasses, however, mowing caused uncertain responses with large variations (Peterson et al., 2013) or a complete failure to change (Wilen and Holt, 1996). However, not all perennial plants responded to the green light by increasing their starch content. For example, the tropical perennial plant *Alpinia oxyphylla* showed lower starch content in shoots under the green light as opposed to the red light (Zhou et al., 2021b). Starch in another temperate perennial plant, *Allium victorialis*, was not affected by different light spectra (Zhou et al., 2021a). Overall, mowing can benefit starch accumulation in alfalfa shoots as a stable effect, but its interaction with light spectra is species-specific.

Accumulating evidence suggests that nonstructural carbohydrate metabolism is a tradeoff between reserve and consumption. During the growing process, when photosynthetic assimilates are continuously used to produce carbohydrates, both coagulation and hydrolyzation occur in carbohydrate granules, and both sugar and starch exist in alternating high/low concentrations (Liu et al., 2021). During consumption, however, starch is intensively depleted for physiological demand while sugars can be accumulated (Wei and Guo, 2017). According to our research, both mowing and the green-light spectrum can increase the content of sugars. These findings suggest that alfalfa seedlings were subjected to a process that reserves photosynthetic production when exposed to green light and mowing. These two treatments did not generate any combined effects on sugar accumulation. The green light likely induced a control on hydrolyzations of both starch and sugars, but it had no further impact on activated conversions.

### Cycling and discrimination of carbon in different isotopes

Mowing resulted in a decline of δ^13^C, suggesting higher conductance and gas exchange. In contrast, on abandoned cropland, it was found that mowing increased the δ^13^C of *Artemisia frigida*, suggesting elevated WUE (Diao et al., 2021). The elevation of δ^13^C is an evolutionary strategy to reserve water loss for a wide spectrum of plant species. However, elevated WUE indicated by δ^13^C is not needed during the evolutionary process for alfalfa. In our study, mown shoots showed higher nonstructural carbohydrates but lower C content. This suggests structural C depletion and likely reserved conversion of structural carbohydrates towards the nonstructural forms. This is evidence of resistance to drought by reserving sugar and starch post-mowing because the depletion of nonstructured carbohydrates is an immediate response to provide energy to fuel enzymic activity (Wei and Guo, 2017; Lauriks et al., 2022).

The elevation of δ^13^C in drought-treated alfalfa correlated with controlled conductance and lowered gas exchange (Erice et al., 2011; He et al., 2020). The depletion of starch was an alternative response to an increase in crude fibers, which further accounted for the increase in total C content. The decrease in sugar content corroborated the significant reduction in gas exchange and intercellular CO_2_ concentration caused by drought (Gorthi et al., 2019; Du et al., 2020). In regard to this study, unchanged sugar content means the close of the stomata did not cause high intercellular CO_2_ to a level that enforced sugar decline. The reduction of starch content in drought is driven to balance the high demands of glucose following limited photosynthesis (Abdelhakim et al., 2021).

Green light induced consistently higher levels of δ^13^C and total C relative to the blue light, which accounts for the above-mentioned increases in DM production and growth. Together with dual increases of soluble sugars and starch contents, it can be concluded that the green light promoted DM accumulation by controlling consumption and accumulating structural and nonstructural carbohydrates. However, these responses to light spectra were interrupted by the mowing. Thus, postmowing differences between δ^13^C and total C content were dismissed. Overall, mowing is the stronger driver compared to light spectra.

### Forage quality and driving forces

Fiber is a type of structural carbohydrate and, following mowing, decreased during the experiment. This result concurs with our hypothesis that fiber was decreased by mowing. Davies et al. (2009) also reported a decrease of crude fibers in mown *Artemisia tridentata* ssp. *wyomingensis*, and they further revealed that the decrease was mainly attributed to the decline of acid detergent fiber. Increased crude fat content in mowed alfalfa was also found in forage bermudagrass (*Cynodon dactylon* [L.] Pers.) (Zhang et al., 2020). Alfalfa is a legume C3 plant, and bermudagrass is a C4 plant dwelling in a warm climate. Both alfalfa and bermudagrass are perennial, and their shoots regrow to stubbles after mowing; their responses of fat accumulation reflect a common physiological consequence of shoot removal. Decreased fiber and increased fat suggest better forage quality in alfalfa. In contrast, drought decreased the forage quality by increasing crude fiber content and decreasing crude fat content. All of these responses were reported in previous studies on grasslands (Grant et al., 2014; Delfani et al., 2022). The lower content of crude fiber in the blue-light spectrum indicates improved forage quality, which, in addition to previous conclusions, demonstrates that blue light limits DM production but improves forage quality.

We found that across treatments, crude fat contents were positively associated with soluble sugar content. Soluble sugars can be dissolved in glycerin because of the formation of hydrogen bonds between glucose and glycerin molecules (van der Sman, 2017). These changes were irrelevant from the exposure to different light spectra because both soluble sugars and crude fat revealed scarce responses to light treatments. DM weight was found to be positively correlated with crude fiber content, which was formed due to dual changes following mowing, drought, light spectra, and their interactions. This means that the increase in DM accumulation is an alternative approach to improving forage quality by increasing fiber content.

## Conclusions

Through a simulated experiment, we found that droughts can interact with the understory light spectrum, which affects DM production in alfalfa. The blue-light spectrum depressed DM production by controlling stomata conductance. Thus, it should be avoided for alfalfa production unless a lower fiber ratio is proposed as the study objective. Mowing can be a reliable approach to activate photosynthetic assimilation/production and improve forage quality by increasing the crude fat ratio and controlling fiber content in alfalfa shoots. Drought depressed DM production and reduced forage quality. Overall, the management of understory alfalfa populations should be considered with forests, where mowing is recommended for alfalfa that are exposed to sunlight transmittance in a higher blue-light spectrum. There, one can expect high-forage quality with lowered crude fibers. In a dry season or when interspecific water competition occurs, irrigation measures will be needed in an agroforestry system to improve alfalfa dry biomass and forage quality.

While we found significant responses of growth, physiology, and forage quality in alfalfa seedlings in this study, all were obtained in a 1-year study, and random factors cannot be fully eliminated from the results. Future studies are encouraged to use more alfalfa genotypes and test the results by cross-year bioassays. Field trials are also suggested for future works to identify indoor findings and to guide agroforestry management with alfalfa as an understory forage species.

## Data availability statement

The original contributions presented in the study are included in the article/Supplementary Material. Further inquiries can be directed to the corresponding authors.

## Author contributions

Conceptualization, CH and JG; methodology, JG; software, YZ; validation, YZ, YW, and JC; formal analysis, CH; investigation, YW; resources, JC; data curation, CH; writing—original draft preparation, CH; writing—review and editing, JZ; visualization, JC; supervision, JZ; project administration, JG; funding acquisition, JG and JZ. All authors have read and agreed to the published version of the manuscript.

## Funding

This research was funded by Fundamental Research Funds for the Central Non-profit Research Institution of CAF (grant number CAFYBB2020SY001 and CAFZC2017M005).

## Conflict of interest

The authors declare that the research was conducted in the absence of any commercial or financial relationships that could be construed as a potential conflict of interest.

## Publisher’s note

All claims expressed in this article are solely those of the authors and do not necessarily represent those of their affiliated organizations, or those of the publisher, the editors and the reviewers. Any product that may be evaluated in this article, or claim that may be made by its manufacturer, is not guaranteed or endorsed by the publisher.
